# A quantum sensor for atomic-scale electric and magnetic fields

**DOI:** 10.1038/s41565-024-01724-z

**Published:** 2024-07-25

**Authors:** Taner Esat, Dmitriy Borodin, Jeongmin Oh, Andreas J. Heinrich, F. Stefan Tautz, Yujeong Bae, Ruslan Temirov

**Affiliations:** 1 Peter Grünberg Institute (PGI-3), Forschungszentrum Jülich, Jülich, Germany; 2 Jülich Aachen Research Alliance (JARA), Fundamentals of Future Information Technology, Jülich, Germany; 3 Center for Quantum Nanoscience (QNS), Institute for Basic Science (IBS), Seoul, South Korea; 4 Department of Physics, Ewha Womans University, Seoul, South Korea; 5 Experimentalphysik IV A, RWTH Aachen University, Aachen, Germany; 6 Faculty of Mathematics and Natural Sciences, Institute of Physics II, University of Cologne, Cologne, Germany; 7 Present Address: Empa, Swiss Federal Laboratories for Materials Science and Technology, nanotech@surfaces Laboratory, Dübendorf, Switzerland

**Keywords:** Nanosensors, Magnetic properties and materials, Scanning probe microscopy

## Abstract

The detection of faint magnetic fields from single-electron and nuclear spins at the atomic scale is a long-standing challenge in physics. While current mobile quantum sensors achieve single-electron spin sensitivity, atomic spatial resolution remains elusive for existing techniques. Here we fabricate a single-molecule quantum sensor at the apex of the metallic tip of a scanning tunnelling microscope by attaching Fe atoms and a PTCDA (3,4,9,10-perylenetetracarboxylic-dianhydride) molecule to the tip apex. We address the molecular spin by electron spin resonance and achieve ~100 neV resolution in energy. In a proof-of-principle experiment, we measure the magnetic and electric dipole fields emanating from a single Fe atom and an Ag dimer on an Ag(111) surface with sub-angstrom spatial resolution. Our method enables atomic-scale quantum sensing experiments of electric and magnetic fields on conducting surfaces and may find applications in the sensing of spin-labelled biomolecules and of spin textures in quantum materials.

## Abstract (web summary)

The fabrication of a molecular quantum sensor on the tip of a scanning tunnelling microscope enables the detection of minute magnetic and electric fields of single atoms with sub-angstrom resolution.

## Main

The spin of an unpaired electron in an atom or molecule, exposed to an external magnetic field, is the quintessential realization of a quantum mechanical two-level system. Consequently, such two-level systems make superb qubits and quantum sensors^1^. Key to both functionalities is the possibility to address the two-level system in an electron spin resonance (ESR) experiment and to read out its spin state. The feasibility of quantum sensors based on ESR has been demonstrated, for example, with nitrogen vacancy (NV) centres in diamond integrated into an atomic force microscope probe tip^2–6^. These sensors have excellent quantum-coherent properties^7^, even at room temperature, and can be optically initialized and read out^2–6^. However, their spatial resolution is limited to tens of nanometres because the sensor must be located ~10 nm inside the diamond and thus atomic resolution has not been achieved^8,9^.

Meanwhile, the functionalization of probe tips with single molecules has expanded the capabilities of scanning probe microscopes. Functionalized tips enable enhanced spatial resolution, for example, to resolve the chemical structure of molecules^10,11^, or new functionalities to image surface potentials^12–14^ and magnetic exchange interactions^15–17^ at the atomic level. However, as the consequence of using non-resonant spectroscopic techniques, their energy resolution is limited by the operating temperature of the scanning tunnelling microscope (STM) to typically ~1.5 meV at 5 K. Moreover, no coherent control of the molecular probes is possible in these approaches^12,13,15–17^. Recently, terahertz spectroscopy enabled coherent control of a hydrogen molecule trapped in the STM cavity and thus allowed to probe the underlying molecule–surface interaction potential^18^.

ESR-STM realizes spin-resonant detection and control of single atoms on surfaces^19–21^. Even the sensing of magnetic moments of individual atoms has been demonstrated using local ESR-active atoms as sensors^22,23^. However, in these experiments, the sensing system is not mobile, limiting its utility in the context of quantum sensing. In addition, all ESR-STM experiments so far have required insulating substrates^24–26^. Here we show that it is possible to engineer a fully integrated, mobile quantum sensor on an STM tip without the need for an insulating film. We achieve the decoupling of the sensing spin from the metal tip by bringing a planar molecule to an upright-standing configuration on the tip, that is, into a configuration where the molecular plane is oriented perpendicular to the surface plane. In this configuration, the coupling of the molecular spin to the metal is reduced, allowing ESR measurements. We demonstrate the sensitivity of the quantum sensor to electric and magnetic fields and derive the corresponding transduction parameters. Finally, we measure the magnetic and electric fields of a single Fe atom and an Ag dimer on an Ag(111) surface with sub-angstrom spatial resolution and quantitatively determine the electric and magnetic dipole moments, which are the sources of these fields.

## Fabrication of the quantum sensor

**Fig. 1:**
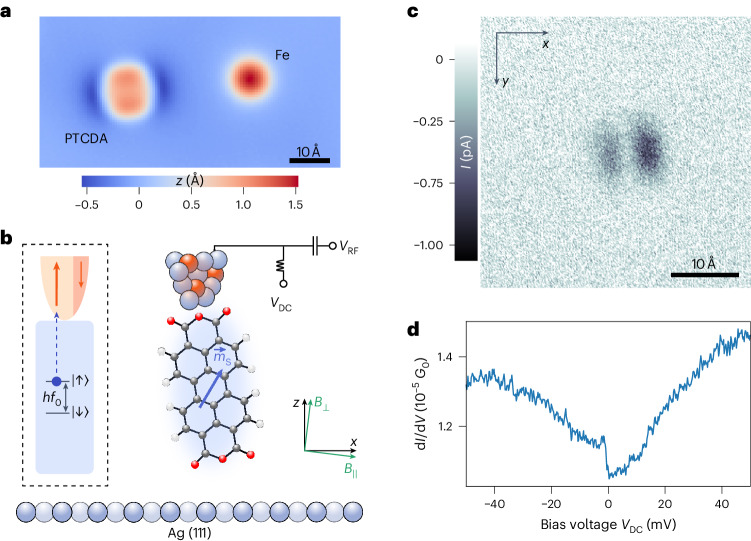
A quantum sensor on the tip of an STM. **a**, Constant-current STM image of the atomic-scale components of the quantum sensor before assembly: a single, flat-lying PTCDA molecule and a single Fe atom on the Ag(111) surface (*V*_DC_ = −300 mV, *I* = 100 pA, imaged with a clean metal tip). **b**, Functional and structural scheme of the assembled quantum sensor. The PTCDA molecule (white, hydrogen; grey, carbon; red, oxygen) is attached in standing configuration to an STM tip, consisting of Ag (light blue) and Fe (orange) atoms. The Fe atoms provide spin polarization. In a ${\vec{B}}$ field (in-plane *B*_||_ and out-of-plane *B*_⊥_ orientations are shown in green), the spin of PTCDA with its magnetic moment ${\vec{m}}_{\text{s}}$ (blue arrow), a quantum two-level system, serves as a sensing qubit. It is addressable by ESR, for which an RF signal *V*_RF_ is added to the DC bias voltage *V*_DC_. The spin-polarized tip reads out the sensor’s quantum state magnetoresistively. **c**, Constant-height STM image of a single Fe atom acquired with the assembled quantum sensor in **b** (*V*_DC_ = −50 mV). Before imaging the Fe atom, the tip with the molecule was stabilized over the bare Ag(111) surface at a setpoint of *V*_DC_ = −50 mV and *I* = 10 pA with feedback turned off, and then retracted by an additional ~2 Å. **d**, d*I*/d*V* spectrum recorded with the quantum sensor over bare Ag(111), exhibiting the signature, that is, an asymmetric step around zero bias, of a spin-½ system measured with a spin-polarized tip^30,31^ (*V*_DC_ = −50 mV, *I* = 50 pA, *V*_mod_ = 1 mV). *G*_0_ = 2*e*^2^/*h* is the quantum of conductance.

We fabricate the quantum sensor in situ from single atoms and a molecule initially adsorbed on the Ag(111) surface (Fig. 1a,b) using the atomic-scale manipulation capabilities of the STM at cryogenic temperatures and ultra-high vacuum conditions^27^ (Methods). We chose a standing PTCDA (3,4,9,10-perylenetetracarboxylic-dianhydride) molecule as the component to introduce the sensing spin. Standing molecules are metal–molecule complexes that can be raised from a flat-lying to a metastable standing configuration, either on the metal surface or on the STM tip^12,28^. A standing PTCDA molecule on the Ag(111) surface is a spin-½ system that is well isolated from the metal surface, as reflected by its very low Kondo temperature of ~290 mK (ref. ^29^). To provide the necessary spin polarization, both for driving and reading out its spin state^19,24^, we augmented the standing PTCDA molecule on the Ag-terminated tip with additional Fe atoms (Fig. 1a,b) that were transferred by voltage pulses from the surface to the tip before attaching the PTCDA to the latter (Methods). An image of a single adatom on the surface, recorded with this probe, confirms the presence of the standing PTCDA molecule on the tip^29^ (Fig. 1c and Methods). Moreover, the differential conductance (d*I*/d*V*, where *I* is the current and *V* is the voltage) spectrum of the probe exhibits the typical signature of a spin-½ system that is measured with a spin-polarized tip^30,31^ (Fig. 1d), namely an asymmetric step at zero bias. This proves that the additional Fe atoms do not alter the spin properties of PTCDA at the tip apex while at the same time giving spin polarization to the tip. Additional studies conducted for standing PTCDA on the surface confirm that the molecule at the tip is not directly bonded to Fe atoms (Methods).

**Fig. 2:**
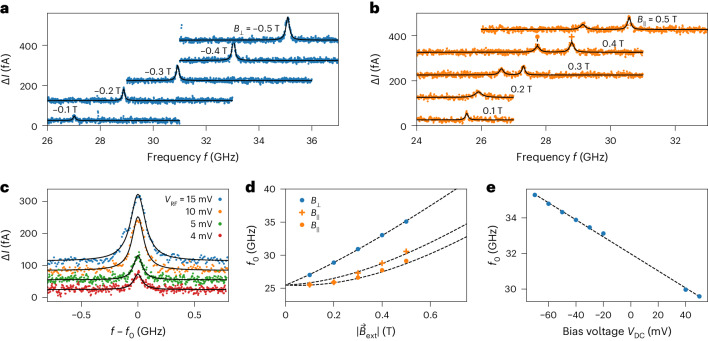
ESR spectra of the quantum sensor. **a**, ESR spectra measured with different *B*_⊥_ on the bare Ag(111) surface (*V*_DC_ = −70 mV, *I* = 5 pA, *V*_RF_ = 15 mV). The current difference Δ*I* results from the different spin state populations of the quantum sensor and the tunnelling magnetoresistance effect during the ESR frequency sweep. The spectra are shifted vertically by multiples of 100 fA for clarity. Black lines are fits with a Lorentzian function. **b**, Same as **a**, but for *B*_||_. **c**, Magnified view of the ESR spectrum at *B*_⊥_ = −0.5 T for different driving amplitudes *V*_RF_ (*V*_DC_ = −70 mV, *I* = 10 pA). Fitting a Lorentzian function yields a full-width at half-maximum of *Γ* ≈ 157 MHz and *Γ* ≈ 93 MHz for *V*_RF_ = 15 mV and *V*_RF_ = 4 mV, respectively. **d**, Resonance frequencies *f*_0_ extracted from **a** and **b** as a function of the magnitude of the external field ${\vec{B}}_{\text{ext}}$. The dashed lines show the calculated *f*_0_ based on the applied external field and the determined tip field. **e**, Dependence of *f*_0_ on *V*_DC_. Corresponding spectra were measured on the bare Ag(111) surface (*I* = 15 pA, *V*_RF_ = 15 mV, *B*_⊥_ = −0.5 T). The dashed line is a linear fit. The standard deviation of the extracted *f*_0_ in **d** and **e** is *σ* ≲ 3 MHz for all data points and smaller than the size of the data point symbols.

We demonstrate that the assembled probe is ESR active and can therefore be used as a quantum sensor. To this end, we add a radio-frequency (RF) voltage *V*_RF_ to the DC bias voltage *V*_DC_ while the probe is located over the bare Ag surface (Methods). Electron spin resonances occur at frequencies *f*_0_, which depend on the magnitude and direction of the applied magnetic field (Fig. 2a–c). We postulate that the applied RF electric field induces a change in the exchange field between the tip and the spin on the molecule. This converts the RF electric field into an RF magnetic field, which, in turn, drives spin transitions between the Zeeman-split $m=\pm \frac{1}{2}$ states of the molecular spin whenever the RF frequency *f* matches the transition energy (*f* = *f*_0_)^32,33^. We note that an alternative origin of the RF driving mechanism could be the piezoelectric coupling of the molecule to the tip^24,34–36^. The change in the spin state of the molecule is detected by the tunnelling magnetoresistance effect stemming from the Fe atoms on the tip, yielding different currents for the two spin directions.

## Response to external fields

**Extended Data Fig. 1:**
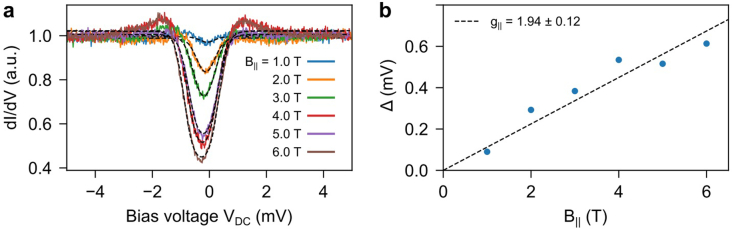
In-plane *g* factor of a standing PTCDA on a Ag + Ag pedestal. **a**, ${\text{d}I}/{\text{d}V}$ spectra measured at different ${B}_{{||}}$ on a standing PTCDA molecule on a Ag + Ag pedestal on the Ag(111) surface (${V}_{\text{DC}}=-5\,\text{mV}$, $I=10\,\text{pA}$, ${V}_{\mathrm{mod}}=0.5\,\text{mV}$). The assembly procedure is described in ref. ^28^. **b**, The Zeeman splitting Δ extracted from the ${\text{d}I}/{\text{d}V}$ spectra in **a** as a function of ${B}_{{||}}$. The black dashed line shows the linear fit for the Zeeman splitting $\Delta ={g}_{{||}}{\mu }_{B}{B}_{{||}}$. The best least-squares fit yields ${g}_{{||}}=1.94\pm 0.12$. The out-of-plane $g$ factor of this structure has been determined in previous work^29^ as ${g}_{\perp }=2.006\pm 0.007$. Note that within experimental error the d*I*/d*V* spectra and the $g$ factors are the same for standing PTCDA molecules on the Ag(111) surface and on the STM tip, as long as the latter are ESR active.

**Extended Data Fig. 2:**
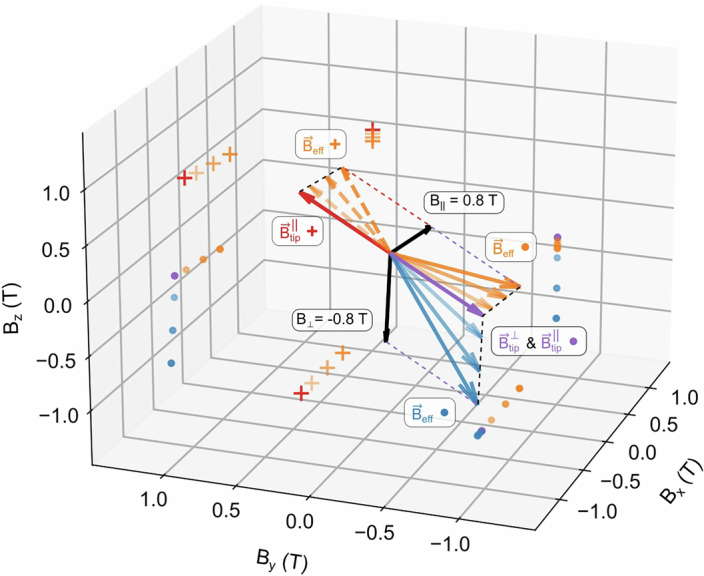
Orientation of the tip field and the quantization axis of the sensor spin. The orientation of the tip field ${\vec{B}}_{\text{tip}}$ is determined from the data in Fig. 2d (Methods) for the out-of-plane ($\perp$, purple) and in-plane (||, purple and red) directions of the external field. For the in-plane direction, ${\vec{B}}_{\text{tip}}$ flips between two orientations (purple and red) that are rotated by 180°, resulting in two resonances for the in-plane (||) direction. The symbols (dot, cross) next to ${\vec{B}}_{\text{tip}}$ and ${\vec{B}}_{\text{eff}}$ indicate the relation to the measured resonances (symbols according to Fig. 2b and d, main text). ${B}_{{||}}$ and ${B}_{\perp }$ (black) denote the externally applied fields, respectively. ${\vec{B}}_{\text{eff}}={\vec{B}}_{\text{tip}}+{\vec{B}}_{\text{ext}}$ is the effective field experienced by the quantum sensor. The orange (blue) arrows show the evolution of ${\vec{B}}_{\text{eff}}$ for increasing ${B}_{{||}}$ (${B}_{\perp }$). The magnetic moment ${\vec{m}}_{\text{s}}={\vec{e}}_{\text{s}}{\mu }_{\text{B}}$ of the sensing spin is always aligned with ${\vec{B}}_{\text{eff}}.$ The dots and crosses on the xy, yz, and xz planes indicate the projection of ${\vec{B}}_{\text{eff}}$, respectively.

**Extended Data Fig. 3:**
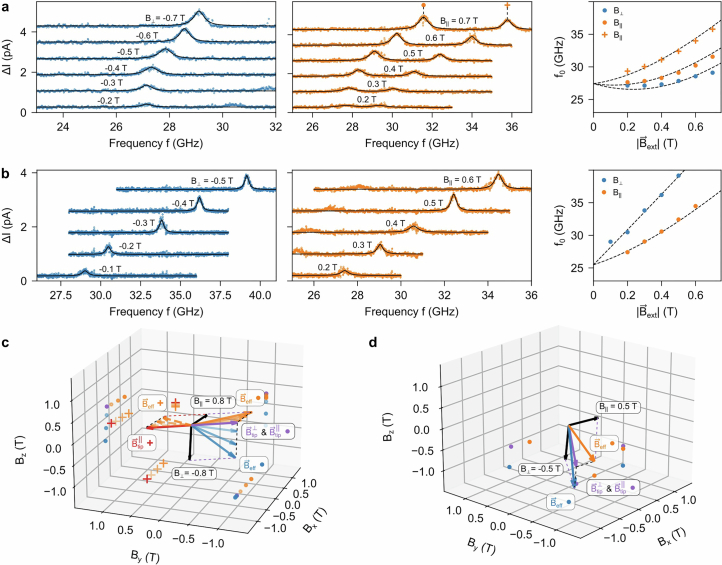
ESR spectra of different quantum sensors. **a**, ESR spectra measured on the bare Ag(111) surface for different ${B}_{\perp }$ (left panel, blue) and ${B}_{{||}}$ (middle panel, orange) with the standing PTCDA molecule attached to the spin-polarized tip (${V}_{\text{DC}}=50\,\text{mV}$, $I=15\,\text{pA}$, ${V}_{\text{RF}}=15\,\text{mV}$). Spectra are shifted vertically for clarity. The resonance frequencies ${f}_{0}$ as a function of the external field $\left|{\vec{B}}_{\text{ext}}\right|$ (right panel). Note that the data were recorded with a different quantum sensor than the one discussed in the main text. The dashed lines show the calculated ${f}_{0}$ based on the applied external field and the determined tip field. **b**, Same as **a**, but for yet another quantum sensor (${V}_{\text{DC}}=-40\,\text{mV}$, $I=15\,\text{pA}$, ${V}_{\text{RF}}=15\,\text{mV}$). All quantum sensors, including the one discussed in the main text, have been fabricated by identical processes. **c**, Orientation of the tip fields for the quantum sensor shown in **a**. The notation is analogous to that of Extended Data Fig. 2. **d**, Same as **c**, but for the quantum sensor shown in **b**.

**Extended Data Fig. 4:**
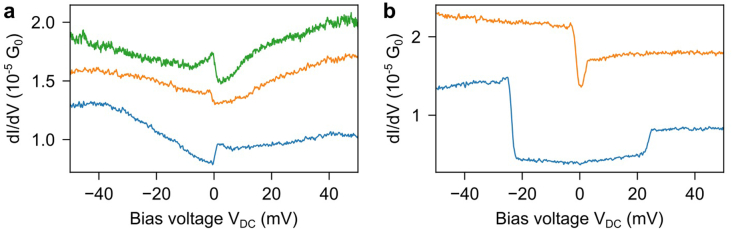
d*I*/d*V* spectra of standing PTCDA molecules on spin-polarized STM tips. **a**, ${\text{d}I}/{\text{d}V}$ spectra recorded with three different fully functional quantum sensors, that is, spin-polarized tips with attached standing PTCDA that are ESR active (${V}_{\text{DC}}=-50\,\text{mV}$, $I=50\,\text{pA}$, ${V}_{\mathrm{mod}}=1\,\text{mV}$). **b**, Typical ${\text{d}I}/{\text{d}V}$ spectra of standing PTCDA on spin-polarized tips that were not ESR active. These assemblies showed inelastic spin excitations with different threshold energies (blue and orange are two examples) in the ${\text{d}I}/{\text{d}V}$ spectrum (${V}_{\text{DC}}=-50\,\text{mV}$, $I=50\,\text{pA}$, ${V}_{\mathrm{mod}}=1\,\text{mV}$). All spectra in **a** and **b** were recorded over bare Ag(111). Spectra are shifted vertically by multiples of $0.25\times {10}^{-5}{G}_{0}$ in **a** and $1.0\times {10}^{-5}{G}_{0}$ in **b** for clarity.

Next, we determine the orientation of the sensor spin’s quantization axis, which is a prerequisite for using it as a ${\vec{B}}$-field sensor. In ESR-STM, the effective field experienced by the quantum sensor is a vector sum of the external field and the tip field (${\vec{B}}_{\text{eff}}={\vec{B}}_{\text{ext}}+{\vec{B}}_{\text{tip}}$). ${\vec{B}}_{\text{tip}}$ can vary in magnitude from a few millitesla to several tesla for atoms on a surface^37^. In our experiment, *f*_0_ does not vanish for ${\vec{B}}_{\text{ext}}\to 0$ (Fig. 2d) but reaches a finite value of ~25.5 GHz. We assign this offset to the tip field ${\vec{B}}_{\text{tip}}$. For a spin-½ system, an offset of ~25.5 GHz at ${\vec{B}}_{\text{ext}}=0\,{\rm{T}}$ corresponds to $\left|{\vec{B}}_{\text{tip}}\right|=\frac{h{f}_{0}}{g{\mu }_{{\rm{B}}}}\approx 0.9\,\text{T}$, which substantially exceeds the external magnetic field, where *μ*_B_ is the Bohr magneton and *h* is the Planck constant. Here we have used the fact that the sensor spin has an isotropic *g* factor of ~2, which we deduced from measurements of its inelastic spin excitations at high *B*_⊥_ and *B*_||_ (Extended Data Fig. 1 and ref. ^29^). In line with previous reports^38^, the dependence of the resonance frequency *f*_0_ on the external field in Fig. 2d can be described by a ${\vec{B}}_{\text{tip}}$ that changes its orientation by 180° in response to ${\vec{B}}_{\text{ext}}$, that is, points up or down along an easy axis while retaining its magnitude. This allows us to determine the easy axis of ${\vec{B}}_{\text{tip}}$ (Methods) and to trace the orientation of the sensor spin’s quantization axis for each ${\vec{B}}_{\text{ext}}$ (Methods and Extended Data Fig. 2). The observation of two resonances for *B*_||_ is thus attributed to a flipping of ${\vec{B}}_{\text{tip}}$ along the easy axis (Extended Data Fig. 2), occurring on a timescale much faster than the ESR frequency sweep^39^. We note that all quantum sensors studied in this work showed a similar behaviour when ${\vec{B}}_{\text{ext}}$ approached 0 T, including the deviation from the linear trend (Extended Data Fig. 3), but deviate from each other with respect to their tip field orientation and bistability (Methods and Supplementary Section 1). Approximately 5% of the fabricated probes with standing PTCDA and Fe atoms exhibited quantum-sensor functionality, that is, could be driven by ESR and read out by tunnelling magnetoresistance (Extended Data Fig. 4a). These tips showed d*I*/d*V* spectra of the type shown in Fig. 1d. All others showed asymmetric inelastic spin excitations around zero bias at energies >|±2| mV in the d*I*/d*V* spectrum (Extended Data Fig. 4b), and no ESR signal was observed.

The quantum sensor is also sensitive to electric fields. The sensitivity to electric fields is not uncommon for quantum sensors^1^; for example, in NV centres^9,40,41^, electric field sensing is based on the Stark effect^42^. Here we attribute this coupling to the deformation of the soft tip–molecule bond by electrostatic forces^29^ and proceed to quantify the strength of the coupling by varying the bias voltage *V*_DC_^43^ and recording the response of the resonance frequency *f*_0_ (Fig. 2e). The transduction parameter *γ* between *f*_0_ and *V*_DC_ at constant height can be determined from the data in Fig. 2e (Methods), yielding *γ* ≈ −0.047 GHz mV^−1^. We note that the observed continuous and unidirectional shift of the resonance frequency for bias voltages of both polarities (Fig. 2e) is direct evidence of coupling to the electric field^36^. If the sensor-surface distance *z* is known, this transduction parameter can also be related to the electric field coupling strength *δ* via *δ* = *γz* and thus directly compared with NV centres. In the normal tunnelling regime, our sensor-surface distance is approximately 15 Å (Methods), corresponding to an electric field coupling strength of *δ* ≈ 66 MHz µm V^−1^, which is ~400 times larger than for NV centres^9^.

On the basis of the above results, the resonance frequency of our quantum sensor is given by

1 $${f}_{0}\left({\vec{r}}\right)=\frac{{g{{\mu }}}_{\mathrm{B}}\left|{\vec{B}}_{\text{eff}}\right|}{h}+\gamma {V}_{\text{DC}}+\frac{\gamma }{\alpha (z)}{\Phi }^{* }\left({\vec{r}}\right)+\frac{1}{h}{E}_{\text{dd}}\left({\vec{r}}\right),$$

where the first two terms account for the Zeeman energy and the effect of the DC bias voltage. The last two terms describe the electric and magnetic dipole moments of a local object, such as an atom on the surface, which may shift the ESR frequency of the quantum sensor. In this equation, ${\Phi }^{* }({\vec{r}})$ is the local object’s electrostatic potential at the position of the sensor, and ${E}_{\text{dd}}({\vec{r}})$ is the magnetic dipole–dipole interaction energy between the magnetic moments of the local object and the sensor spin. Here $\vec{r}=(x,y,z)$ is the position of the sensor with respect to the local object, and *α*(*z*) accounts for the fact that ${\Phi }^{* }({\vec{r}})$ is a potential difference between the sensor and the sample surface, while the bias voltage is applied between the tip and the sample (Methods).

## Sensing electric and magnetic fields from single atoms

**Fig. 3:**
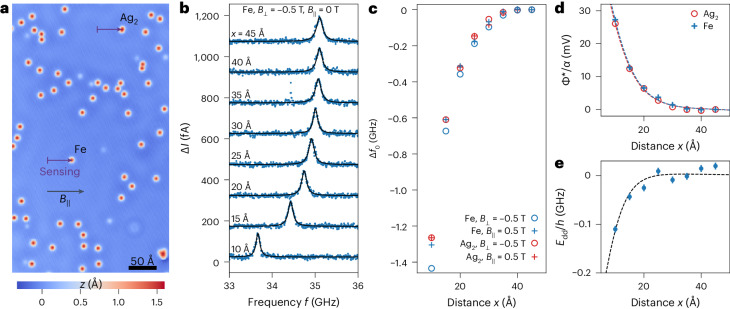
Measuring electric and magnetic dipole moments of atomic objects. **a**, Constant-current STM image of Fe atoms and an Ag dimer on the Ag(111) surface measured with an Ag-terminated metal tip. The paths along which ESR was recorded with the mobile quantum sensor and the in-plane direction of *B*_||_ are indicated by the respective arrows (*V*_DC_ = −300 mV, *I* = 100 pA). **b**, ESR spectra recorded with *B*_⊥_ = −0.5 T along the lower path indicated in **a** for different distances *x* from the Fe atom (*V*_DC_ = −70 mV, *I* = 5 pA, *V*_RF_ = 15 mV). Spectra are shifted vertically by multiples of 150 fA for clarity. Black lines are fits with a Lorentzian function. **c**, Resonance frequency shift Δ*f*_0_ of the quantum sensor in response to the local electric and magnetic fields originating from the Ag dimer and the Fe atom, plotted as a function of distance *x* to the respective object. External fields: *B*_⊥_ = −0.5 T or *B*_||_ = 0.5 T as indicated. The standard deviation of Δ*f*_0_ is *σ* ≲ 5 MHz for all data points and smaller than the size of the data point symbols. **d**, Electrostatic potential ${\Phi }^{* }/\alpha =\Delta {f}_{0}/\gamma$ for the Ag dimer and Fe atom. Data points are extracted from **c** and the standard deviations of ${\Phi }^{* }/\alpha$ are *σ* ≲ 0.2 mV and smaller than the size of the symbols. The dashed lines are fits of a point-dipole potential using the normalized PSF ${\xi }^{* }$ of the quantum sensor (Methods). The fit yields an electric dipole moment of ${P}_{\perp ,{\text{Ag}}_{2}}=0.84\pm 0.02\,\text{D}$ for the Ag dimer and ${P}_{\perp ,\text{Fe}}=0.89\pm 0.02\,\text{D}$ for the Fe atom. **e**, Magnetic interaction energy *E*_dd_/*h* of the Fe atom. The dashed line is a fit of the analytical expression (equation (3)) yielding a magnetic dipole moment of ${|\vec{m}}_{\text{Fe}}|=3.2\pm 0.4{\mu }_{\text{B}}$. The standard deviations of *E*_dd_/*h* are *σ* ≲ 7 MHz for all data points. The additional scatter in the data points may be related to an uncertainty in the lateral positioning in *x* (<0.1 Å) between the out-of-plane and in-plane measurements.

We now use the quantum sensor to determine the electric and magnetic dipole moments of atomic objects on the Ag(111) sample surface. For this purpose, a circular area of about 100 Å diameter around the target object (either an Ag dimer or a single Fe atom) was cleared by lateral manipulation with the STM tip to avoid parasitic effects of unwanted objects (Fig. 3a). The quantum sensor was then moved towards the target object, while ESR spectra were recorded at a fixed setpoint as a function of the lateral distance *x* (Fig. 3b). To exclude additional effects caused by the magnetic exchange interaction^23,44^ between target and sensor, the experiments were performed at distances ≥10 Å. The measurements were performed for two different field orientations, namely *B*_⊥_ = −0.5 T and *B*_||_ = 0.5 T. To eliminate the effects owing to ${\vec{B}}_{\text{eff}}$ and *V*_DC_, we subtract *f*_0_ at *x* = 45 Å, that is, far from the target objects, from the resonance frequencies ${f}_{0}\left({\vec{r}}\right)$ because the influence of the electric and/or magnetic dipole moments on the sensor at these distances is negligible. We thus obtain

2 $$\Delta {f}_{0}\left({\vec{r}}\right)\equiv {f}_{0}\left({\vec{r}}\right)-{f}_{0}\left(x=45\,{{\text{\AA }}},y,\,z\right)=\frac{\gamma }{\alpha \left(z\right)}{\Phi }^{* }\left({\vec{r}}\right)+{\frac{1}{h}E_{\rm{dd}}}\left({\vec{r}}\right)$$

for the shift of the resonance frequency. Figure 3c shows the shift of the resonance frequency when either the Ag dimer or the Fe atom is approached. There is a characteristic difference between approaching Fe and Ag_2_ on the surface: in the case of Ag_2_, there is no dependence on the external magnetic field, while in the case of Fe, we observe a clear difference between the measurements with *B*_⊥_ and *B*_||_. Evidently, this difference arises from the interaction between the sensor spin and the magnetic moment of the Fe atom, which is not present in the case of non-magnetic Ag_2_. Thus, dividing the measured $\Delta {f}_{0}\left({\vec{r}}\right)$ for Ag_2_ by the transduction parameter *γ* directly yields ${\Phi }^{* }\left({\vec{r}}\right)/\alpha (z)$ (Fig. 3d). Assuming that Ag_2_ is represented well by a point dipole and fitting the data in Fig. 3d with a normalized point spread function (PSF) that also takes the screening of the dipole’s potential by the metal tip into account (Methods), we dispense with the unknown function *α*(*z*) and obtain an electric dipole moment of ${P}_{\perp ,{\text{Ag}}_{2}}=0.84\pm 0.02\,\text{D}$ for Ag_2_. This result is in excellent agreement with the recent independent measurement of ${P}_{\perp ,{\text{Ag}}_{2}}^{\,{\text{SQDM}}}=0.95\pm 0.1\,\text{D}$ obtained with scanning quantum dot microscopy (SQDM)^45^. We note, however, that our ESR-based detection scheme with its large *γ* has a factor of ~20 higher electric field sensitivity than SQDM at the same operating temperature of 1.4 K, because the field sensitivity of SQDM is limited by temperature.

**Extended Data Fig. 5:**
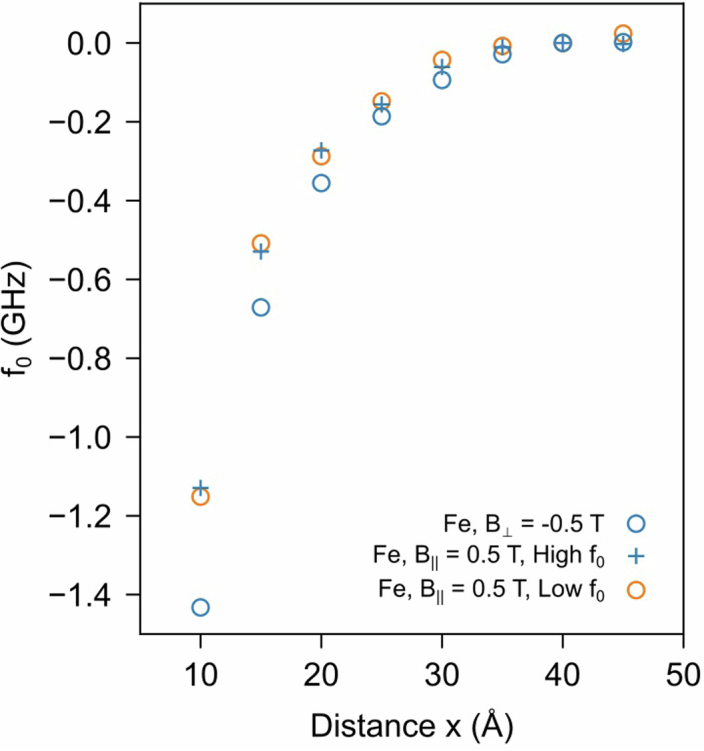
Sensing of the electric and magnetic dipole moment of Fe. Resonance frequency shift $\Delta {f}_{0}$ of the quantum sensor in response to the local electric and magnetic fields originating from the Fe atom, plotted as a function of distance $x$ (see also Fig. 3a). The two resonances (blue cross and orange circle) of the quantum sensor for ${B}_{{||}}$ (see also Fig. 2b and d) shift identically when the Fe atom is approached, indicating a vanishing magnetization of Fe for in-plane magnetic fields. Symbols and colours according to Fig. 3c.

To exemplify the power of the quantum sensor, we simultaneously determine the electric and magnetic dipole moments of the Fe atom on the surface. The Fe atom on Ag(111) has a strong easy-axis magnetic anisotropy along the out-of-plane direction and shows paramagnetic behaviour^46^, which means that its intrinsic moment is only measurable in a saturating external field. We find that the two observed resonances for *B*_||_ (Fig. 2b), which correspond to the two different orientations of the sensor spin’s quantization axis (Extended Data Fig. 2), shift identically when the Fe atom is approached (Extended Data Fig. 5). Because an out-of-plane magnetic moment of the Fe atom would interact differently with the sensor spin in its two orientations, thereby yielding different frequency shifts, we conclude that for the in-plane measurements, the magnetization of the Fe atom is vanishingly small (${|{\vec{m}}}_{\text{Fe}}|\approx 0$) and below our detection limit (effectively ${E}_{\text{dd}}\left({\vec{r}}\right)\approx 0$ in equation (2)). Therefore, the frequency shifts at *B*_||_ = 0.5 T are only sensitive to the electric dipole contributions, that is, ${\Delta f}_{0}^{\,{||}}\left({\vec{r}}\right)=\frac{\gamma }{\alpha \left(z\right)}{\Phi }^{* }\left({\vec{r}}\right)$. Following the same procedure as for Ag_2_, we obtain an electric dipole moment of *P*_⊥,Fe_ = 0.89 ± 0.02 D for the Fe atom on Ag(111) (Fig. 3d).

In the out-of-plane field direction, the electric and magnetic dipole contributions in equation (2) are superimposed and need to be disentangled. Since the effect of the Fe atom’s electric potential on Δ*f*_0_ is independent of ${\vec{B}}_{\text{ext}}$, we can eliminate this contribution to the shift of the ESR by calculating the difference (Fig. 3e)

3 $${\Delta f}_{0}^{\,\perp }\left({\vec{r}}\right)-{\Delta f}_{0}^{\,||}\left({\vec{r}}\right)={E}_{\text{dd}}^{\perp }\left({\vec{r}}\right)/h.$$

This quantity captures the magnetic dipole–dipole interaction between the magnetic moments of the quantum sensor and Fe atom. We fit the experimental data in Fig. 3e with equation (3) and find ${|{\vec{m}}}_{\text{Fe}}|=3.2\pm 0.4{\mu }_{\text{B}}$ (Methods). This agrees well with the literature report of ~3.1 ± 0.1 *μ*_B_ for Fe on the Ag(111) surface^46^, suggesting that it reaches magnetic saturation at ~0.5 T, consistent with other magnetic atoms on metals^47^.

## Spatial resolution

To quantify the spatial resolution of the quantum sensor, we examined the shift of the resonance frequency *f*_0_ as a function of the position *x* during the lateral approach of the Fe atom in the sensing experiments shown in Fig. 3. The resonance frequency shift in Fig. 3b is approximately 760 MHz between the measurements taken at 10 Å and 15 Å, and the ESR peak has a full-width at half-maximum of 120 MHz. Thus, the ESR peak shifted by ~6.3 peak widths over a distance of 5 Å, implying a spatial resolution of at least ~0.8 Å. Note that this is an upper bound for the spatial resolution since the resonance frequency *f*_0_ can be determined with high accuracy even if the shift is less than one peak width. With standard peak fitting methods, *f*_0_ can be determined with an accuracy of at least 0.2 peak widths, which in our experiment yields a lateral resolution of <0.2 Å.

## Conclusion

**Extended Data Fig. 6:**
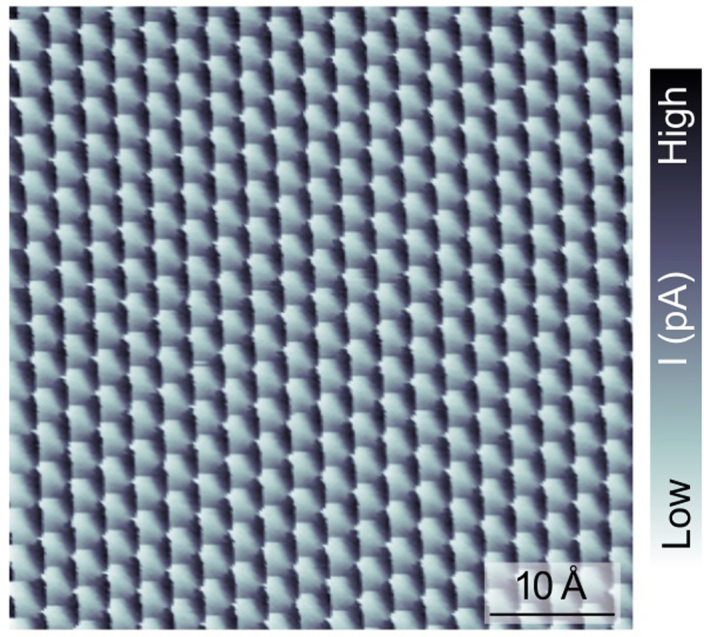
Atomically resolved Ag(111) surface imaged with the quantum sensor. Constant-height STM image (${V}_{\text{DC}}=-1\,\text{mV}$). The setpoint before scanning the image was ${V}_{\text{DC}}=-1\,\text{mV}$, *I* = 100 pA. Immediately before recording the image, the assembly of the quantum sensor was completed by pulling the PTCDA molecule, initially flat on the surface, vertically up by ~12 Å.

We have fabricated and used a fully integrated, mobile quantum sensor for the simultaneous measurement of atomic-scale electric and magnetic fields. It is assembled from atomic-scale components on the tip of an STM and combines an energy resolution of ~100 neV with sub-angstrom spatial resolution. The superior energy resolution of our sensor compared with non-resonant spectroscopic techniques in STM is defined by the narrowness of the detected ESR, which, in turn, is determined by the spin relaxation and decoherence times of the sensor spin. Thus, even in a continuous-wave experiment, our quantum sensor benefits from the coherence of the ESR process. Importantly, the quantum sensor can also be used in conventional STM mode to image atoms, molecules and nanostructures with atomic resolution (Fig. 1c and Extended Data Fig. 6). This allows the precise positioning of the quantum sensor on any surface that is amenable to imaging with STM, which is beneficial for subsequent sensing studies. The concept of the quantum sensor relies on the decoupling of a molecular spin from the metallic STM tip, by a carefully designed bonding of the molecule to the latter, overcoming the need to use thin insulating layers for spin decoupling in ESR-STM. We anticipate that this quantum sensor can also be used as a mobile spin qubit, which can be coupled to other atomic-scale qubits on surfaces^21^. To fully exploit its potential, a promising next step will be the combination with pulsed ESR techniques. Finally, we anticipate that single-molecule quantum sensors, such as the one developed in this work, will become of interest for biochemistry, where the electronic structure of catalytic centres in enzymes and other biomolecules on metal surfaces^48^ can now be revealed with atomic-scale precision.

## Methods

### Sample preparation

The Ag(111) surface was prepared in ultra-high vacuum by repeated cycles of sputtering with Ar^+^ and heating to 800 K, and then transferred to the cold STM. The individual Fe atoms and PTCDA molecules were deposited in situ on the sample at approximately 10 K. To allow in situ deposition of atoms and molecules on the sample inside the STM, the sample is not aligned with the axis of the magnet^27^. As a consequence, the two perpendicular components (in-plane and out-of-plane) of the vector magnet are tilted ~7° with respect to the sample surface plane (Fig. 1b).

### STM and ESR measurements

All experiments were performed at 1.4 K in a home-built STM with a two-axis vector magnet and ESR capability with a wide frequency range^27^. Differential conductance (d*I*/d*V*) spectra were measured using the conventional lock-in technique with the feedback loop switched off and an a.c. modulation amplitude *V*_mod_. The PtIr tip was treated in situ by controlled voltage pulses and indentations into the clean Ag surface, resulting in a clean Ag-coated tip. The spectroscopic signature of the Ag(111) surface state was used to confirm the cleanliness of the tip. The frequency-dependent transmission losses of the cables in the STM were compensated by adjusting the source power of the RF signal generator during the sweeps of the RF frequency *f* to obtain a constant amplitude *V*_RF_ at the tunnel junction for the continuous-wave ESR experiments^49^. All ESR spectra were measured in constant-current mode with very low feedback gain to compensate for slow drift and to keep the tip–sample distance constant.

### Fabrication of the quantum sensor

The quantum sensor was constructed in two steps. First, a spin-polarized STM tip was fabricated by transferring up to three Fe atoms from the Ag(111) surface to the Ag-coated non-magnetic tip apex. To transfer individual Fe atoms from the Ag(111) surface to the tip, a voltage pulse of 1.6–1.9 V was applied while the tip was withdrawn from near point contact with the Fe atom. Second, one of the four carboxylic oxygen atoms of an isolated PTCDA molecule on the surface was contacted with the tip, whereupon a covalent bond between the tip apex and the oxygen atom formed^50,51^. In the next step, the tip with the attached molecule was pulled vertically upwards by about 12 Å until the molecule was completely detached from the surface and reached the standing orientation on the tip. This procedure decouples the PTCDA molecule from the metal and results in a singly charged radical^28,29,50–53^. Confirmation of the standing orientation came, first, from imaging the thus completed quantum sensor by scanning it across a single Fe atom on the surface—the Fe atom acts as an effective tip on the surface to scan the real tip apex, that is, the standing PTCDA molecule. The resulting image in Fig. 1c with its two elliptical features resembles the STM image of a standing PTCDA molecule on a pedestal of two Ag atoms on the Ag(111) surface^28,29^. This proves the presence of a standing PTCDA molecule in the fabricated quantum sensor. Second, after its fabrication, the quantum sensor was used to record an atomically resolved image of the Ag(111) surface (Extended Data Fig. 6). The tip height at which this image was scanned proves the presence of a standing molecule of approximately 12 Å height above the metallic tip apex.

### Structure of the quantum sensor

**Extended Data Fig. 7:**
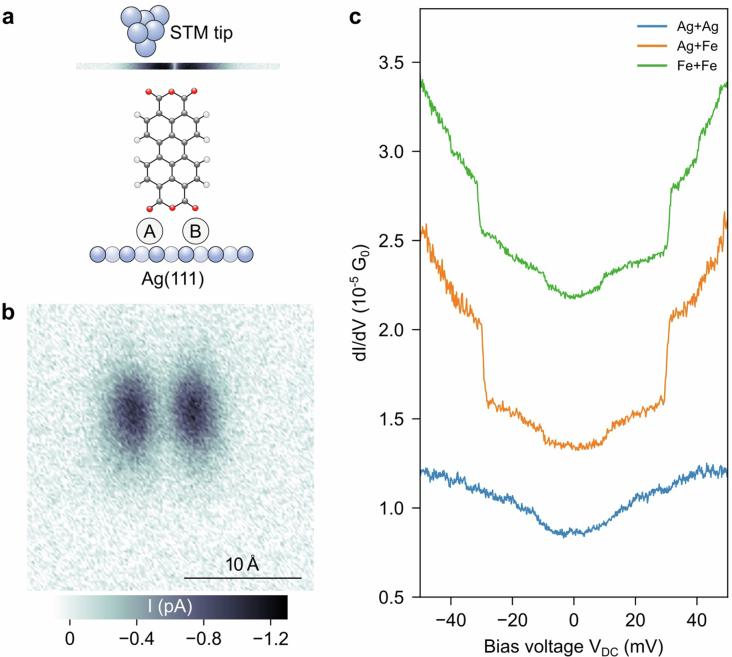
PTCDA molecules standing on a pedestal of different metal adatoms on Ag(111). **a**, Schematic structure of the standing metal-molecule structure including the STM tip above the PTCDA molecule. The bar shows the tunnel current $I$ measured above the structure at constant height. The pedestal atoms A and B are placeholders for either Ag or Fe atoms. **b**, Typical constant-height STM image over the standing molecule on a Ag+Ag, Ag+Fe and Fe+Fe pedestal, recorded at a tip height of $z\simeq 18$ Å above the surface (${V}_{\text{DC}}=-50\,\text{mV}$). The fabrication procedure for the different standing structures is analogous to the one for PTCDA on the Ag+Ag pedestal^28^. **c**, ${dI}/{dV}$ spectra measured on one of the lobes of the standing metal-molecule structure on a Ag+Ag (blue, ${V}_{\text{DC}}=-50\,\text{mV}$, $I=50\, \text{pA}$, ${V}_{\mathrm{mod}}=1.0\,\text{mV}$), Ag+Fe (orange, ${V}_{\text{DC}}=-60\,\text{mV}$, $I=65\,\text{pA}$, ${V}_{\mathrm{mod}}=0.5\,\text{mV}$) and Fe + Fe (green, ${V}_{\text{DC}}=-50\,\text{mV}$, $I=50\,\text{pA}$, ${V}_{\mathrm{mod}}=0.5\,\text{mV}$) pedestal. Spectra are shifted vertically by multiples of 1.0 × 10^−5^*G*_0_ for clarity.

To determine the structure of the quantum sensor at the base of the standing molecule, that is, at the interface to the metallic STM tip, we constructed various standing molecules on the Ag(111) and measured their d*I*/d*V* spectra. Comparing these with the d*I*/d*V* spectra of the quantum sensor, we obtained information about the structure and composition of the quantum sensor. Specifically, we placed the PTCDA molecule on Ag + Fe and Fe + Fe pedestals on the Ag(111) surface by controlled manipulation with the STM tip (Extended Data Fig. 7). The procedure is analogous to fabricating standing PTCDA on Ag + Ag pedestals on Ag(111)^28^. If the pedestal of standing PTCDA on the Ag(111) surface contained at least one Fe atom, inelastic spin excitations at higher voltages were observed in the d*I*/d*V* spectrum (Extended Data Fig. 7), similar to those at the tip when PTCDA is not ESR active (Extended Data Fig. 4). Therefore, we conclude that the ESR-active PTCDA on the tip, that is, the PTCDA in the functioning quantum sensor, cannot be directly bound to an Fe atom, but must be standing on an Ag + Ag pedestal, which is consistent with its d*I*/d*V* spectrum that is characteristic of a spin-½ system. The Fe atoms on the tip thus only enable the ESR driving mechanism and provide the spin polarization for the detection of the spin state of the quantum sensor^24,34,35^, but are not directly bound to the standing molecule. The Ag + Ag pedestal on the Ag tip donates an electron into the standing PTCDA, thus providing the sensing spin ½.

### Magnetic coupling within the quantum sensor

A relatively large ${\vec{B}}_{\text{tip}}$ with a magnitude of ~0.9 T (main text) is generated as a result of magnetic interactions between Fe atoms on the tip and the sensing spin. Magnetic couplings giving rise to local ${\vec{B}}$ fields of this magnitude are typically generated by neighbouring atoms at distances of ~2 Å via direct exchange^37,44^. At first sight, this suggests a direct binding of PTCDA to at least one of the Fe atoms on the tip, but this can be ruled out as discussed above. Therefore, we speculate that the magnetic coupling between the Fe atoms and the sensor spin is mediated by the Ruderman–Kittel–Kasuya–Yosida interaction, which is known to reach sufficient strength in similar atomic arrangements^54^. In this model, the Fe atoms at the tip induce a spin polarization in the conduction electrons of neighbouring Ag atoms of the Ag-coated tip, which, in turn, polarize the sensor spin, thereby generating the large ${\vec{B}}_{\text{tip}}$ at the sensor spin. The polarization of the Ag conduction electrons also explains the sensitive detection of the quantum spin state by tunnelling magnetoresistance.

### Orientation of the quantization axis of the sensing spin

The effective field ${\vec{B}}_{\text{eff}}=({B}_{x}+{T}_{x},{B}_{y}+{T}_{y},{B}_{z}+{T}_{z})$ experienced by the quantum sensor is a vector sum of the known external field ${\vec{B}}_{\text{ext}}=({B}_{x},{B}_{y},{B}_{z})$ and the initially unknown tip field ${\vec{B}}_{\text{tip}}=({T}_{x},{T}_{y},{T}_{z})$. Since the sensor is a spin-½ system (isotropic *g* factor ≈ 2; main text), the magnetic moment ${\vec{m}}_{\text{s}}={\vec{e}}_{\text{s}}{\mu }_{\text{B}}$ of the sensing spin is aligned with ${\vec{B}}_{\text{eff}}$, that is,

4 $${\vec{e}}_{\text{s}}=\frac{{\vec{B}}_{\text{eff}}}{\left|{\vec{B}}_{\text{eff}}\right|}.$$

The orientation and strength of ${\vec{B}}_{\text{tip}}$ can be deduced from the magnetic field dependence of the resonance frequency *f*_0_ of the sensor (Fig. 2d), analogous to previous STM-based ESR experiments^38^. It is generally assumed that Fe-decorated magnetic tips have large magnetic anisotropies and that a change of the external magnetic field direction may cause the tip field to flip by 180° along an easy axis^38^.

According to equation (1), in the absence of electric and magnetic dipole moments of a local object, the magnetic-field-dependent resonance frequency ${f}_{0}({\vec{B}}_{\text{eff}})$ is related to $\left|{\vec{B}}_{\text{eff}}\right|$ by

5 $$|\,{\vec{B}}_{{\rm{eff}}}|=\sqrt{{({B}_{x}+{T}_{x})}^{2}+{({B}_{y}+{T}_{y})}^{2}+{({B}_{z}+{T}_{z})}^{2}}=h\,\frac{{f}_{0}({\vec{B}}_{{\rm{eff}}})-\gamma {V}_{{\rm{DC}}}}{g{\mu }_{{\rm{B}}}}.$$

In our experimental setup, the components of ${\vec{B}}_{\text{ext}}$ are given by *B*_*x*_ = *B*_||_ cos θ + *B*_⊥_ sin *θ*, *B*_*y*_ = 0 and *B*_*z*_ = −*B*_||_ sin *θ* + *B*_⊥_ cos *θ* with *θ* = 7° (main text and Fig. 1a). To determine the three unknown vector components *T*_*x*_, *T*_*y*_ and *T*_*z*_ of ${\vec{B}}_{\text{tip}}$, we fit equation (5) simultaneously to all recorded resonance frequencies *f*_0_ for the different out-of-plane (⊥) and in-plane (||) external fields (Fig. 2d), allowing for a 180° flip of the tip field orientation between the two detected resonances for the in-plane direction. From the fit, we find that the resonances for out-of-plane (blue dots in Fig. 2d) and at low frequency for in-plane (orange dots in Fig. 2d) result from the same orientation of the tip field. The resonances at high frequency for in-plane (orange crosses in Fig. 2d) result from the tip field orientation being flipped by 180°. Finally, the orientation of the sensor spin ${\vec{e}}_{\text{s}}$ follows from ${\vec{B}}_{\text{eff}}$ according to equation (4). The resulting orientations of ${\vec{B}}_{\text{tip}}$, ${\vec{B}}_{\text{eff}}$ and ${\vec{e}}_{\text{s}}$ for the magnetic field dependencies of *f*_0_ shown in Fig. 2d are shown in Extended Data Fig. 2.

Note that the orientation of ${\vec{B}}_{\text{tip}}$ strongly influences *f*_0_ and its response to the external magnetic fields. Therefore, it is not possible to deduce the orientation of ${\vec{B}}_{\text{tip}}$ without fitting the model to the data. We have also applied the above tip model to the two quantum sensors shown in Extended Data Fig. 3 and obtained a good agreement between the experimental data and the model. This confirms the general applicability of our model to describe the behaviour of the quantum sensors in the external magnetic field. It is also possible to rationalize the bistability of ${\vec{B}}_{\text{tip}}$ and thus the occurrence of two ESR lines once its orientation has been determined (Supplementary Section 1).

### Transduction parameter

To characterize the transduction parameter *γ* for electric field sensing, we varied the bias voltage *V*_DC_ and recorded the response of the resonance frequency *f*_0_ at a fixed setpoint current of *I* = 15 pA. Since these measurements were performed in constant-current mode, the tip–sample distance also varied as the bias voltage was changed. Note that a change in the tip–sample distance will result in a different electric field felt by the sensor, since to a first approximation (plate-capacitor model) the electric field *E* in the STM junction is related to the distance *z* by *E* = *V*_DC_/*z*. To disentangle the effects of the bias voltage *V*_DC_ and the change in distance on the response of *f*_0_, we performed two additional experiments.

**Extended Data Fig. 8:**
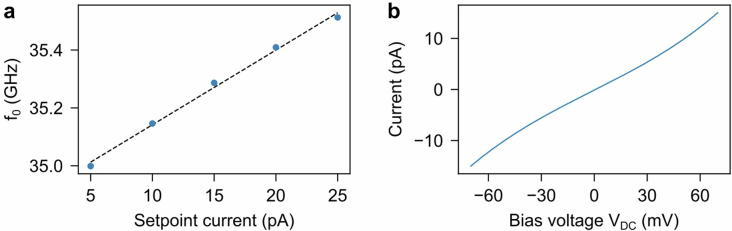
Data for relative tip-sample distance calibration of ESR experiments. **a**, Dependence of ${f}_{0}$ on the setpoint current $I$ for a constant bias voltage. Corresponding spectra were measured on the bare Ag(111) surface (${V}_{\text{DC}}=-70\,{\rm{mV}}$, ${V}_{\text{RF}}=15\,\text{mV}$, ${B}_{\perp }=-0.5\,\text{T}$). Same quantum sensor as used for the sensing experiments in the main text. **b**, Constant-height $I(V)$ spectrum recorded with the quantum sensor over bare Ag(111) surface. Initial setpoint was ${V}_{\text{DC}}=-70\,\text{mV}$, $I=15\,\text{pA}$.

First, we measured the dependence of *f*_0_ on the setpoint current *I* for a fixed bias voltage of *V*_DC_ = −70 mV in constant-current mode, that is, with the feedback on (Extended Data Fig. 8a). In this case, the change in *f*_0_ is solely the result of a change in the tip–sample distance, since the feedback loop regulates the distance to reach the setpoint current for the fixed bias voltage. In the measured range from 5 pA to 25 pA, we find a linear dependence of *f*_0_ on *I*. A linear fit to the data in Extended Data Fig. 8a yields a slope of 25.8 MHz pA^−1^. Second, we recorded an *I*(*V*) spectrum with the quantum sensor in constant-height mode, that is, at fixed tip–sample distance (Extended Data Fig. 8b). For this measurement, the tip was initially stabilized at one of the setpoints used for the measurement of the bias dependence of *f*_0_, that is, *V*_DC_ = −70 mV, *I* *=* 15 pA (Fig. 2e). These two data sets allowed us to correct for the effect of the distance change when measuring *f*_0_ as a function of *V*_DC_ in constant-current mode.

To illustrate this, we focus on measurements at negative bias voltages (Fig. 2e), for the sake of simplicity. Extended Data Fig. 8b reveals that changing the bias voltage from −70 mV to −20 mV would change the current from 15 pA to 6 pA if we were measuring in constant-height mode. However, since during the ESR sweep at 15 pA we are in constant-current mode, the feedback loop decreases the tip–sample distance to correct for the 9 pA current reduction at constant height between the two bias voltages. According to Extended Data Fig. 8a, this 9 pA reduction corresponds to an additional resonance frequency shift of ~−230 MHz. The resonance shift from −70 mV to −20 mV is −2.163 GHz at constant current (Fig. 2e). Corrected for the effect of the distance decrease (current increase), the resonance frequency shift is therefore −2.393 GHz at constant height.

To obtain the transduction parameter *γ*, we corrected all *f*_0_ that were measured as a function of *V*_DC_ at constant current for the effect of the distance/current change, as described above, and fitted the resulting data with a linear fit, yielding −0.047 GHz mV^−1^ (Fig. 2e). Notably, the linearity of the *f*_0_ versus *V*_DC_ curve in Fig. 2e is essentially preserved when data are plotted at constant height, because the *I*(*V*) dependence is close to linear for the low bias voltages considered here (cf. Extended Data Fig. 8b).

We also determined the transduction parameters for the sensors in Extended Data Fig. 3a,b, obtaining *γ* ≈ *−*0.065 GHz mV^−1^ and *γ* ≈ −0.104 GHz mV^−1^, respectively. Note that these values are not corrected for changes of the tip–sample distance.

### Electric dipole moments

According to the Helmholtz equation

6 $${\Phi }_{{\rm{s}}}({\vec{r}}^{\,\prime} )=\frac{1}{{{\epsilon }}_{0}}{\Pi }_{\perp }({\vec{r}}^{\,\prime} ),$$

the surface potential ${\Phi }_{\text{s}}$ is related to the perpendicular dipole density ${\Pi }_{\perp }$. The task of determining the (perpendicular) electric dipole moments

7 $${P}_{\perp }={\iint_{{\rm{surface}}}}{\Pi }_{\perp }({\vec{r}}^{\,\prime} ){d}^{2}{\vec{r}}^{\,\prime} \approx {\Pi }_{\perp }({\vec{r}}^{\,\prime} )A$$

of the Ag dimer and the Fe atom thus reduces to measuring the surface potential of the sample (*A* is the area covered by the dimer or atom, respectively). Since the quantum sensor responds to the electric potential ${\Phi }^{* }$ at the position $\vec{r}$ of the sensor spin, that is, at the position of the PTCDA molecule on the tip, the task amounts to reconstructing the surface potential ${\Phi }_{\text{s}}\left(\vec{r}{\prime} \right)$ in the object surface from the potential ${\Phi }^{* }(\vec{r})$ in the imaging plane that is located several Å above the object surface. ${\Phi }_{\text{s}}\left(\vec{r}{\prime} \right)$ and ${\Phi }^{* }(\vec{r})$ are related by an electrostatic boundary value problem. It was shown that their relation can be expressed as^55^

8 $${\Phi }^{\ast }({\vec{r}})={\iint_{{\rm{surface}}}}\xi ({\vec{r}},{\vec{r}}^{\,\prime} ){\Phi }_{{\rm{s}}}({\vec{r}}^{\,\prime} ){d}^{2}{\vec{r}}^{\,\prime} ,\,$$

where the PSF $\xi$ can be calculated from the Dirichlet Green’s function. If the tip–sample junction is approximated by parallel plates (pp), equation (8) becomes

9 $${\Phi }^{\ast }({\vec{r}})=\,{\iint_{{\rm{surface}}}}{\xi }_{{\rm{pp}}}\left(|{\vec{r}}_{\parallel }-{\vec{r}}_{\parallel }^{\,\prime} |,z\,\right){\Phi }_{{\rm{s}}}({\vec{r}}_{\parallel }^{\,\prime} ){d}^{\,2}{\vec{r}}_{\parallel }^{\,\prime} ,$$

with $${\vec{r}}=({\vec{r}}_{||},{\rm{z}})$$.

If a point dipole is placed at ${\vec{r}}_{\parallel }^{\,\prime}$ on the planar surface, the surface potential will develop a deformation

10 $${\Phi }_{{\rm{s}}}({\vec{r}}^{\,\prime\prime} )=\frac{{P}_{\perp }}{{{\epsilon }}_{0}}\delta \left({\vec{r}}_{||}^{\,\prime\prime} -{\vec{r}}_{||}^{\,\prime} \right),$$

where ${\epsilon }_{0}$ is the vacuum permittivity, and one obtains from equation (9)

11 $${\Phi }^{\ast }({\vec{r}})=\frac{{P}_{\perp }}{{{\epsilon }}_{0}}{\xi }_{{\rm{pp}}}\left(|{\vec{r}}_{\parallel }-{\vec{r}}_{\parallel }^{\,\prime} |,z\right)$$

for the potential at the position of the sensor. This is the potential that changes the ESR frequency. We calculated ${\Phi }^{* }\left(\vec{r}\right)$ by adding up the potentials of the original dipole and an infinite series of image dipoles that are generated by alternately mirroring at the planar tip and sample surfaces, thus taking the screening of the dipole’s field by both the metallic sample and the metallic tip into account^55^. This screening increases the lateral resolution of the quantum sensor because it leads to an exponential decay of the PSF with lateral distance from the sources (here point dipole) in the surface plane.

The sensitivity of the ESR frequency to electric potentials follows from the experimental transduction relation ${f}_{0}=\gamma {V}_{\text{DC}}+{g\mu }_{\text{B}}\left|\,{\vec{B}}_{\text{eff}}\right|/h$ (Fig. 2e), measured on the bare surface (${\Phi }_{{\rm{s}}}=0$) at sufficiently large lateral distance from either the Fe atom or the Ag dimer. At the applied bias voltage *V*_DC_, the potential at the sensor in this case is ${\Phi }_{\text{sensor}}=\alpha {V}_{\text{DC}}$, with $\alpha \equiv d\Phi /d{V}_{{\rm{DC}}}$. Hence, in terms of the acting potential ${\Phi }_{\text{sensor}}$, the transduction relation becomes

12 $${f}_{0}=\left(\frac{\gamma }{\alpha }\right){\Phi }_{{\rm{sensor}}}+g{\mu }_{{\rm{B}}}|\,{\vec{B}}_{{\rm{eff}}}|/h.$$

It should be noted that both *α* and *γ* are properties of the sensor itself and thus may vary for different sensors.

We now return to the case of the point dipole on the surface. On the basis of equations (11) and (12), it causes a frequency shift of the quantum sensor relative to equation (12) of

13 $$\Delta {f}_{0}({\vec{r}})=\left(\frac{\gamma }{\alpha (z)}\right)\frac{{P}_{\perp }}{{{\epsilon }}_{0}}{\xi }_{{\rm{pp}}}\left(|{\vec{r}}_{||}-{\vec{r}}_{||}^{\,\prime} |,z\right).$$

We note that for a general boundary value problem

14 $${\iint_{{\rm{surface}}}}\xi ({\vec{r}},{\vec{r}}^{\,\prime} ){d}^{2}{\vec{r}}^{\,\prime} =\alpha ({\vec{r}})$$

holds^55^. In the present case, this becomes (with equation (11))

15 $$\begin{array}{c}{\displaystyle\iint_{{\rm{surface}}}}{\xi }_{{\rm{pp}}}\left(|\,{\vec{r}}_{||}-{\vec{r}}_{||}^{\,\prime} |,z\right){d}^{\,2}{\vec{r}}_{\parallel }^{\,\prime} ={\displaystyle\iint_{{\rm{imaging}}\,{\rm{plane}}}}{\xi }_{{\rm{pp}}}\left(|{\vec{r}}_{||}-{\vec{r}}_{||}^{\,\prime} |,z\right){d}^{\,2}{\vec{r}}_{||}\\ \,={\displaystyle\iint_{{\rm{imaging}}\,{\rm{plane}}}}\frac{{{\epsilon }}_{0}}{{P}_{\perp }}{\Phi }^{\ast }\left({\vec{r}}_{||},z\right){d}^{\,2}{\vec{r}}_{||}=\,\alpha (z)\,\end{array}.$$

If one normalizes the function $\frac{{\epsilon }_{0}}{{P}_{\perp }}{\Phi }^{* }({\vec{r}}_{\parallel },z)$ that was calculated by summing up the infinite series of image dipole potentials (see above) to yield 1 after integration over the imaging plane at each *z*, a normalized PSF ${\xi }_{\text{pp}}^{\,* }\left(\left|{\vec{r}}_{\parallel }-{\vec{r}}_{\parallel }^{\prime} \right|,z\right){=\xi }_{\text{pp}}\left(\left|{\vec{r}}_{\parallel }-{\vec{r}}_{\parallel }^{\prime} \right|,z\right)/\alpha (z)$ is obtained with which equation (13) finally becomes

16 $$\frac{\Delta {f}_{0}({\vec{r}})}{\gamma }=\frac{{P}_{\perp }}{{{\epsilon }}_{0}}{\xi }_{{\rm{pp}}}^{\,\ast }\left(|\,{\vec{r}}_{||}-{\vec{r}}_{||}^{\prime} |,z\right).$$

The unknown *α*(*z*) thus drops out.

We used equation (16) to fit the experimentally measured ${\Delta f}_{0}\left(\vec{r}\right)/\gamma$ data for the Ag dimer and the Fe atom (Fig. 3d,e). To this end, ${\xi }_{\text{pp}}^{\,* }\left(\left|{\vec{r}}_{\parallel }-{\vec{r}}_{\parallel }^{\prime} \right|,z\right)$ functions for each *z* were precalculated in steps of 0.5 Å. Then, *P*_⊥_ and *z* were used as fit parameters, thereby selecting the pair that yields the smallest *χ*^2^ error. We note that two identical values for the heights, *z*_Ag_ = 14.5 ± 1.0 Å for Ag_2_ and *z*_Fe_ = 14.5 ± 1.0 Å for Fe, were obtained in the two independent fits of the Ag_2_ and Fe data that were recorded at the same tip heights. This confirms the reliability of the procedure. Note that *z* reflects the height of the imaging (sensing) plane, not the height of the tip.

### Magnetic dipole moments

The magnetic dipole–dipole interaction between the magnetic moment ${\vec{m}}_{\text{s}}$ of the quantum sensor and the local magnetic moment ${\vec{m}}_{\text{Fe}}$ of the Fe atom is given by

17 $${{{E}}}_{{\rm{dd}}}({{\vec{r}}})=\frac{{\mu }_{0}}{4\pi {|\,{\vec{r}}|}^{\beta }}[({\vec{m}}_{{\rm{Fe}}}\cdot {\vec{m}}_{{\rm{s}}})-3({\vec{m}}_{{\rm{Fe}}}\cdot {\hat{r}})({\vec{m}}_{{\rm{s}}}\cdot {\hat{r}})]$$

with *β* ≈ 3, where *μ*_0_ is the vacuum permeability, $\vec{r}=(x,y,z)$ is the distance between the two magnetic moments and $\hat{r}$ is the corresponding unit vector. The vertical distance *z* between the two magnetic moments stems from the height difference between the PTCDA molecule (sensor) on the tip and the Fe atom on the surface and thus indicates where the magnetic sensing occurs in the molecule (Supplementary Section 2). We assume that the electric and magnetic sensing take place at the same point in the molecule, that is, we set *z* = *z*_Fe_ = 14.5 Å as obtained from the determination of the electric dipole moment (Methods). The orientation of the quantization axis of the quantum sensor and its magnetic moment is extracted from the magnetic field dependence in Fig. 2d (Methods). It is ${\vec{m}}_{\text{s}}\approx (-0.1,-0.6,-0.8){\mu }_{\text{B}}$ for *B*_⊥_ = −0.5 T in Fig. 3c. Owing to the uniaxial magnetic anisotropy of Fe on Ag(111)^46^, only the out-of-plane component *m*_Fe,*z*_ of the magnetic moment of Fe is considered, that is, *m*_Fe,*x*_ = *m*_Fe,*y*_ = 0. Finally, to fit the analytical expression ${{{E}}}_{{\rm{dd}}}(\vec{{{r}}})$ (equation (17)) to the data in Fig. 3e, we use *m*_Fe,*z*_ and *β* as free fit parameters. The best least-squares fit yields *m*_Fe,*z*_ = −3.2 ± 0.4 *μ*_B_ and *β* = 3.1 ± 0.05. The fitted *β* is in good agreement with the characteristic exponent (*β* ≡ 3) of the magnetic dipole–dipole interaction, confirming the assumption that the interaction originates from the dipole–dipole interaction. Moreover, the excellent agreement between the magnetic moment of Fe determined with the quantum sensor and the literature value (main text) confirms the assumption that the electric and magnetic sensing occur at the same location in the sensor (Supplementary Section 2). We note that the quantum sensor is not restricted to sensing out-of-plane components, but can be used as a three-dimensional $\vec{B}$-field sensor, allowing the determination of magnetic moments with arbitrary orientation.

**Extended Data Fig. 9:**
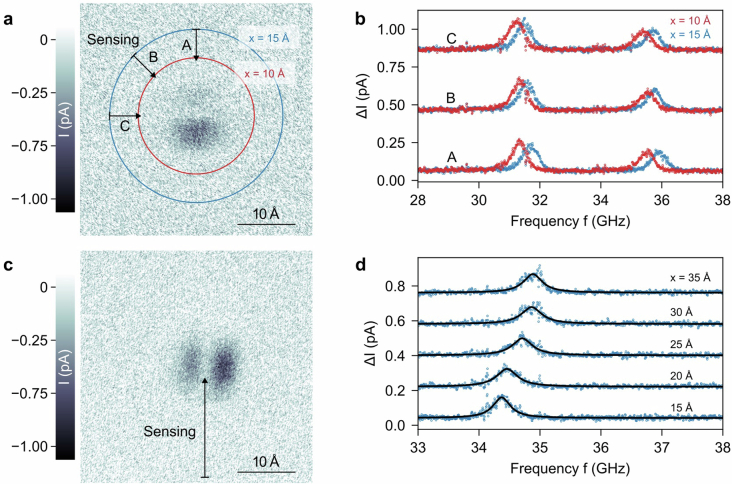
Sensing with different quantum sensors. **a**, Constant-height STM image of a single Fe atom acquired with the quantum sensor presented in Extended Data Fig. 3a (${V}_{\text{DC}}=-50\,\text{mV}$). The arrows indicate the paths along which ESR spectra were recorded with the mobile quantum sensor. **b**, ESR spectra recorded with ${B}_{{||}}=0.7\,\text{T}$ for the paths indicated in **a** for different distances $x$ from the Fe atom (${V}_{\text{DC}}=50\,\text{mV}$, $I=15\,\text{pA}$, ${V}_{\text{RF}}=15\,\text{mV}$). Spectra are shifted vertically by multiples of 400 fA for clarity. **c**, As **a**, but for the quantum sensor presented in Extended Data Fig. 3b. **d**, ESR spectra recorded with ${B}_{\perp }=-0.35\,\text{T}$ for the path indicated in **c** for different distances *x* from the Fe atom (${V}_{\text{DC}}=-40\,\text{mV}$, $I=15\,\text{pA}$, ${V}_{\text{RF}}=15\,\text{mV}$). Spectra are shifted vertically by multiples of 180 fA for clarity.

We note that for all fabricated quantum sensors, we observed a response to the local electric and magnetic fields of an Fe atom. The resonance frequency response for the sensors presented in Extended Data Fig. 3 is shown in Extended Data Fig. 9 for different lateral approach directions.

## Online content

Any methods, additional references, Nature Portfolio reporting summaries, source data, extended data, supplementary information, acknowledgements, peer review information; details of author contributions and competing interests; and statements of data and code availability are available at 10.1038/s41565-024-01724-z.

## Supplementary information

Supplementary Information Supplementary Sections 1 and 2.

## Data Availability

All data presented in this study are available on the Jülich Data Repository at 10.26165/JUELICH-DATA/ZSSLXL.
