# Supplementary material for: A quantum sensor for atomic-scale electric and magnetic fields
Source: Nat Nanotechnol. 2024 Jul 25;19(10):1466–71. doi: 10.1038/s41565-024-01724-z (PMC11486657; doi:10.1038/s41565-024-01724-z)
Supplement: Supplementary file 1 — Supplementary Sections 1 and 2. [file 41565_2024_1724_MOESM1_ESM.pdf]

nature nanotechnology

Supplementary information https://doi.org/10.1038/s41565-024-01724-z

A quantum sensor for atomic-scale electric and magnetic fields

In the format provided by the authors and unedited

Table of Contents

Supplementary Section S1.	Bistability of the tip magnetic field
Supplementary Section S2.	Sensing position within the quantum sensor

Supplementary Section S1. Bistability of the tip magnetic field

For all functional quantum sensors reported in this work, we have reconstructed the tip field $\vec{B}_{\text{tip}}$ from the detailed functional behaviour of f_0 vs. $B_\perp$ and $B_{||}$ (Fig. 2 and Ext. Data Fig. 2, and Ext. Data Fig. 3). Once the tip field orientation is known, the observed bistabilities can be qualitatively understood. For the sensor in Fig.2 and Ext. Data Fig 2, the tip field's easy axis has almost no component in the x direction, but roughly comparable components in y and z directions. An external field (predominantly) in the x direction (i.e., the applied in-plane field) will therefore stabilize neither of the two possible orientations along the easy axis, with the result that a switching bistability is observed. In contrast, an external field (predominantly) in the z direction (i.e., out-of-plane field) will stabilize one of the two tip-field orientations, such that no switching occurs. This agrees precisely with our experimental observation of two ESR resonances for in-plane and only one for out-of-plane external fields (Fig. 2).

In comparison to the previously discussed sensor, the sensor in Ext. Data Fig. 3a/c has a very small z component of the tip field. Therefore, the easy axis is now almost parallel to the y axis, with the result that neither an in-plane nor an out-of-plane field leads to an appreciable stabilization of one of the two possible orientations along the easy axis. Consequently, bistability is expected for both applied field directions. Again, this is what is observed in experiment, if we note the weak second resonance at higher frequencies for -0.2 T and -0.3 T out-of-plane fields (for higher fields, it falls out of the measured range).

Finally, the sensor in Ext. Data Fig. 3b/d has its easy axis approximately in the z direction (the y component is almost zero, the x component is small). Here we would therefore expect strong stabilization and no bistability for out-of-plane applied fields, and weak stabilization and bistability for in-plane applied fields, once more in agreement with the experimental observations (note the weak resonance at lower frequencies for in-plane fields in Ext. Data Fig. 3b).

Supplementary Section S2. Sensing position within the quantum sensor

It is an interesting question where precisely in the composite quantum sensor, consisting of the metal tip with its Fe atoms and the standing PTCDA molecule, the sensing occurs. Our simple (and as it turns out a posteriori: *accurate*) models assume well-defined sensing heights above the sample surface and moreover suggest that the electric and magnetic field sensing occur at similar heights.

When fitting the electric field data, the sensing height is a fit parameter. Remarkably, we find that both the Fe and Ag_2 yield the same height, namely 14.5 Å. This indicates that the sensing height for the electrical potential is indeed well-defined. Moreover, the agreement with the dipole measured by SQDM shows that our fit model (which assumes a well-defined sensing height) is realistic.

For SQDM, the sensing height was determined from distance-dependent measurements and comparison with simulations that the electric field must be sensed close the centre of the molecule (see ref. [12]). However, since the SQDM-based electric field sensing and ESR-based electric field sensing rely on different physical mechanisms (quantum dot charging vs. straining a soft bond), it is not necessarily the case that the electric field sensing in SQDM and ESR occur at the same sensing heights.

For the magnetic sensing, we imposed in the fit the same sensing height (14.5 Å) as for the electric sensing, initially for simplicity. However, the excellent results for the magnetic dipole of Fe *a posteriori* confirm this choice, because the obtained magnetic dipole depends sensitively on the sensing height (as does the electric dipole: since both fields are power functions of the distance $\boldsymbol{r}$, even small deviations from the correct assumption lead to discrepancies in the obtained electric and magnetic moments). We therefore conclude that electric and magnetic sensing indeed occur at equal (or at least very similar) similar heights.

Having established a sensing height of 14.5 Å above the surface for both electric and magnetic sensing, the position of this point within the probe (metal tip plus standing PTCDA molecule) could in principle be determined by an accurate measurement of the absolute height of the probe above the surface. Such a measurement can principally done by AFM. However, since our experiments have been done in a STM without AFM functionality, such a measurement was not possible in our case.

Short of an accurate absolute height measurement, we can estimate the sensing height based on the STM tunnelling currents. PTCDA has a length of ~15 Å. If the sensing occurred at the interface between the standing PTCDA and the metal tip, the lower end of PTCDA would be in contact with the metal surface. This can be excluded on two grounds: First, the measured currents would be much larger, and second, the sensor would be subjected to lateral distortions, for which we find no evidence in our data. Our tunnel currents suggest distances of 3 to 7 Å between the sample surface and the lower end of the PTCDA molecule, corresponding to a sensing height 2.5 to 6.5 Å below the interface between tip and sensor molecule. To conclude, the common height for electric and magnetic sensing must be located a few Å below the metal tip, i.e., in the upper half of the standing PTCDA molecule.

Finally, we stress that the question of where exactly the sensing occurs is only of secondary importance to our paper, since irrespective of the details, our paper demonstrates an approximately *hundredfold* increase of the lateral resolution compared to the best competing technique (NV centers, ref. [8]), precisely because the sensing occurs so close (within a few Angströms) to the outermost apex of the tip.
